# *Solms-laubachia garzeensis* (Brassicaceae), a new species from the Hengduan Mountains of Sichuan Province, China

**DOI:** 10.3897/phytokeys.273.177843

**Published:** 2026-04-15

**Authors:** Jie Fu, Yu Ma, Zhongyi Zhao, Hongling Chen, Hongxiang Yin

**Affiliations:** 1 College of Ethnomedicine, Chengdu University of Traditional Chinese Medicine, Chengdu 611137, China College of Ethnomedicine, Chengdu University of Traditional Chinese Medicine Chengdu China; 2 School of Pharmacy, Chengdu University of Traditional Chinese Medicine, Chengdu 611137, China School of Pharmacy, Chengdu University of Traditional Chinese Medicine Chengdu China

**Keywords:** Alpine subnival belt, critically endangered, new species, *Solms-laubachia garzeensis, Solms-laubachia* Muschl

## Abstract

A new species of the genus *Solms-laubachia* was discovered in the Hengduan Mountains region of Sichuan Province, China, and it has been described and illustrated. Morphologically, it is similar to *Solms-laubachia himalayensis* (Cambess.) J. P. Yue, Al-Shehbaz & H. Sun, *Solms-laubachia stewartii* (T. Anderson) J. P. Yue, Al-Shehbaz & H. Sun, *Solms-laubachia linearis* (N. Busch) J. P. Yue, Al-Shehbaz & H. Sun. However, by synthesizing the characteristics of simple trichomes, blue-violet petals, large siliques, toothed bracteal leaves and cryptic coloration possessed by the new species, it can be distinctly distinguished from the aforementioned three species. Molecular phylogenetic analysis based on the nuclear ribosomal internal transcribed spacer (*nrITS*) of 23 other species in the same genus also supports the conclusion that this is a new species. Scanning electron microscopy (SEM) of its pollen revealed that it is tricolpate. *Solms-laubachia garzeensis* J. Fu, Y. Ma & H. X. Yin is currently distributed only in the Shaluli Mountains at the junction of Garze County and Xinlong County in Garze Prefecture, Sichuan Province. According to the classification criteria of the International Union for Conservation of Nature (IUCN), it is currently assessed as “critically endangered” (CR).

## Introduction

The Hengduan Mountains (HDM) in southwestern China represent one of the world’s 34 recognized biodiversity hotspots (Sun et al. 2017). This region inhabits a remarkable array of endemic taxonomic groups, including various members of the genus *Solms-laubachia* Muschl. (Xu et al. 2014). *Solms-laubachia* (Brassicaceae) is primarily distributed across the Himalayan-Hengduan Mountains, with the HDM region serving as the center of its species diversity and genetic variation (Yue et al. 2009a; Al-Shehbaz 2015). Based on Bayesian relaxed molecular clock dating, *Solms-laubachia* is inferred to have originated in Central Asia during the Pliocene and subsequently migrated eastward into the Himalayan-Hengduan Mountains (Yue et al. 2009b).

The genus was established by Muschler in 1912, with *S. pulcherrima* from Yunnan Province, China designated as the type species. It has since undergone several taxonomic revisions (Botschantsev 1955; Lan and Cheo 1981; Al-Shehbaz and Yang 2001). A preliminary morphological and molecular systematic study (Yue et al. 2006) revealed that four species of Desideria and *Phaeonychium jafrii* were nested within the *Solms-laubachia* clade. Subsequent research led to the merging of multiple species from these three genera, giving rise to the concept of an “expanded *Solms-laubachia*” (Yue et al. 2009a; German and Al-Shehbaz 2010). The currently delimited *Solms-laubachia* is a well-established monophyletic group comprising 32 species (Chen 2019). Species within this expanded genus typically inhabit the distinctive environment of the alpine subnival belt and are characterized by a low-growing habit, leaves with entire or toothed margins, fruits that dehisce easily and split into an inverted “V” shape, and strikingly colorful flowers in shades of blue, pink, red, and purple.

In the HDM region, *Solms-laubachia* often appears in high-altitude scree slopes and cliff habitats, exhibiting a typical “sky island” distribution pattern. These areas are difficult to access due to extreme elevations, rugged terrain, deep river valleys, and poor road infrastructure. Its unique topography and climate have promoted high rates of speciation and endemism. In recent years, several new species of *Solms-laubachia* have been reported from the HDM (Yue et al. 2005; Yue et al. 2009a; Chen et al. 2018). Each member’s habitat is restricted to its type locality.

During a field survey around HDM in May 2023, a distinctive population of *Solms-laubachia* was discovered on a scree slope at 4700 m elevation. The herb attracted our attention for its distinctive blue-violet petals, nevertheless its leaves possessed unique cryptic coloration harmonizing with the rock background, which is morphologically distinct from all known species of *Solms-laubachia* in HDM. After examining relevant literature, physical specimens, and digitized collections, we concluded that this entity represents a previously undescribed species. It shows some extent similarity with *S. himalayensis*, *S. stewartii*, and *S. linearis*. However, a comprehensive analysis of morphological traits including trichomes, bracts, floral characteristics and silique morphology reveals distinct differences between them.

Subsequently, in 2023, 2024, and 2025, further investigations were conducted in the distribution area of this population and the surrounding regions. A comprehensive morphological description was carried out. The pollen characteristics were observed by scanning electron microscope (SEM). Phylogenetic studies were conducted using the nuclear gene (*nrITS*) sequence. These studies aimed to determine whether genetic differentiation existed between this species and other species within *Solms-laubachia*, and to explore its taxonomic position within the genus *Solms-laubachia*.

## Materials and methods

### Morphological assessment

The morphological measurements and descriptions of the new species were based on living plants in nature and specimens. Some morphological features were observed and measured by means of a stereomicroscope (GP 45T1-530H, Suzhou, China). This study examined specimen collections at PE, KUN, IBSC, CDBI, and HNWP via on-site inspections. It also consulted digital specimens from more than 30 herbaria including K, PE, P, USNM, W, E, MW, and HUN via the Global Biodiversity Information Facility (**GBIF**, https://www.gbif.org/) and the Chinese Virtual Herbarium (**CVH**, https://www.cvh.ac.cn/).

### Pollen grain scanning electron microscopy (SEM)

Mature pollen of *S. garzeensis* was placed on a carrier stage affixed with conductive adhesive, sprayed with gold, observed and photographed by SEM (ZEISS EVO10), and the polar axis length (P) and equatorial axis length (E) were determined, and the pollen polar-eccentric ratio (P/E) was calculated.

### DNA extraction, amplification, sequencing and molecular phylogenetic analysis

Using the method of Carey et al. (2023), total DNA was extracted from freshly dried silica-gel-protected leaves. PCR was performed according to Yue (2006), and the modified PCR primer ITS-18F was used for amplification (White et al. 1990; Mummenhoff et al. 1997). The PCR products were sent to Sangon Biotech (Shanghai, China) for sequencing.

Following the method of Chen et al. (2018), the ribosomal internal transcribed spacer (*nrITS*) was selected as the marker, and sequences of 23 other species in the same genus were downloaded from Genbank. *Christolea crassifolia* and *Leiospora pamirica* were selected as outgroups for molecular phylogenetic analysis.

The molecular phylogenetic analysis was performed using PHYLOSUITE v.1.1.15 (Zhang et al. 2020). Sequence alignment was conducted with MAFFT implemented in PHYLOSUITE and subsequently adjusted manually, with gaps treated as missing data. Maximum Likelihood (ML) analysis was performed using IQ-TREE as implemented in PHYLOSUITE v.1.1.15, with the TMP model selected as the best-fit substitution model by ModelFinder, with branch support evaluated using 1000 ultrafast bootstrap replicates. Bayesian Inference (BI) was carried out using the SYM model, with two independent Markov Chain Monte Carlo (MCMC) runs each set for 10 million generations, sampling every 1000 generations. The first 25% of sampled trees were discarded as burn-in, and a majority-rule consensus tree was generated from the remaining trees to calculate posterior probabilities. The final phylogenetic tree was visualized and annotated using iTOL v.6.6 (https://itol.embl.de).

## Result

### Morphological characters

The new species is morphologically most similar to *S. himalayensis* and *S. stewartii*. It can be easily distinguished from *S. himalayensis* by its toothed bracteal leaves, blue-violet petals, and siliques and seeds twice as long. The trichomes of *S. stewartii* are stalked and exist in both simple and forked forms, which is significantly different from the new species that only has simple trichomes. Meanwhile, the seeds and siliques of the new species are 2 to 3 times larger than those of *S. stewartii*, making it easily distinguishable from *S. stewartii*. The plants of *S. linearis* are smaller in size, and its siliques and seeds are also significantly smaller than those of the new species. Moreover, its purplish-red flowers are strikingly different from the blue-violet petals of the new species.

**Figure 1.**
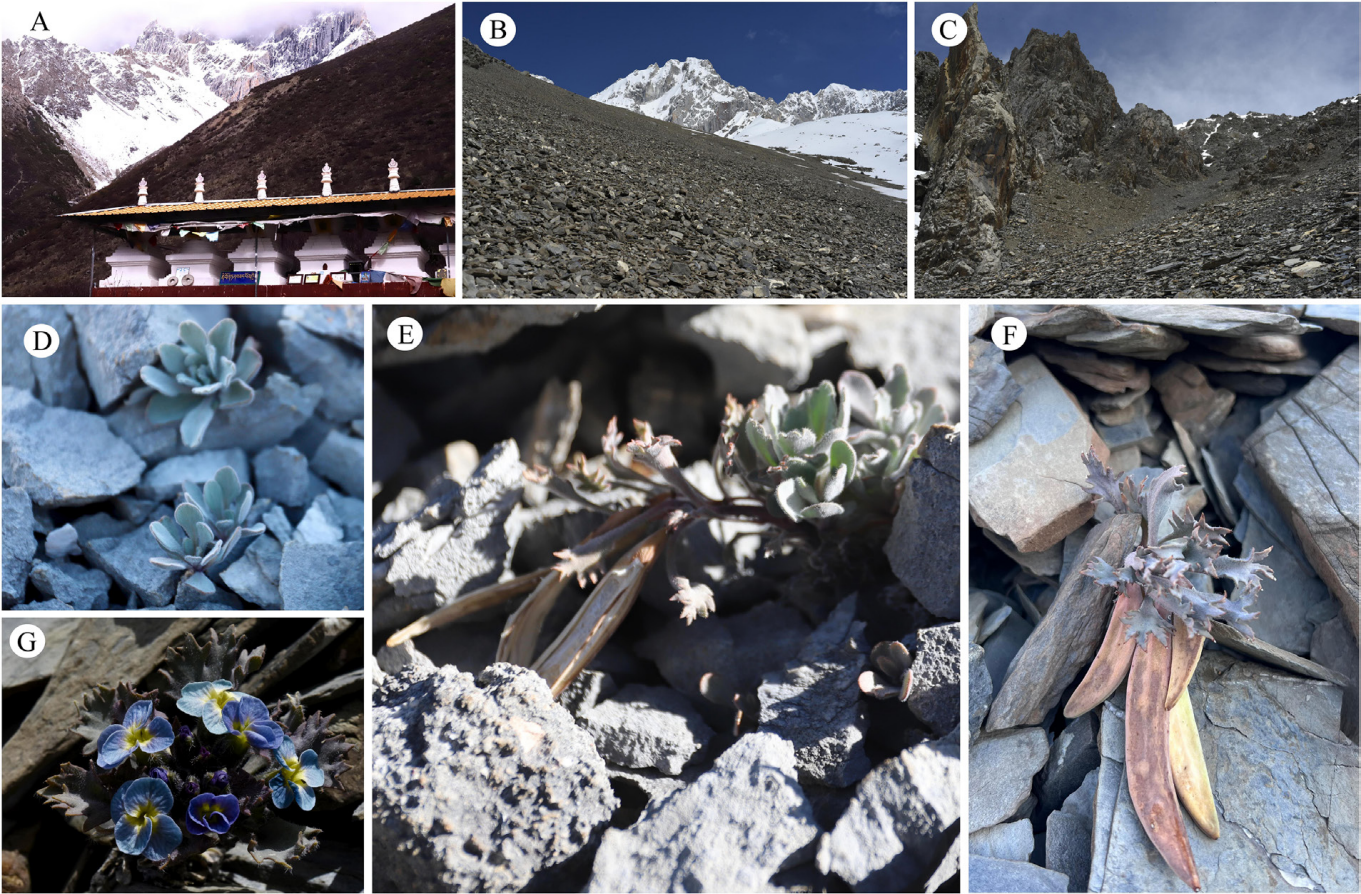
**A**. Shaluli Mountains; **B, C**. Habitat; **D, E**. Plants: Growing on gray limestone scree, leaves nearly gray; **F, G**. Plants: Growing on brownish-gray scree, leaves brownish-gray.

*S. garzeensis* exhibits cryptic coloration that varies depending on its habitat, allowing it to harmonize with surrounding rocks. For instance, individuals growing on gray limestone screes often develop gray leaves (Fig. 1D, E), while those inhabiting tan-colored rocky substrates tend to display brownish foliage (Fig. 1F, G). This adaptive camouflage makes *S. garzeensis* typically difficult to be sought out. This feature also distinguishes it from other species such as *S. himalayensis*, *S. stewartii*, and *S. linearis*.

**Figure 2.**
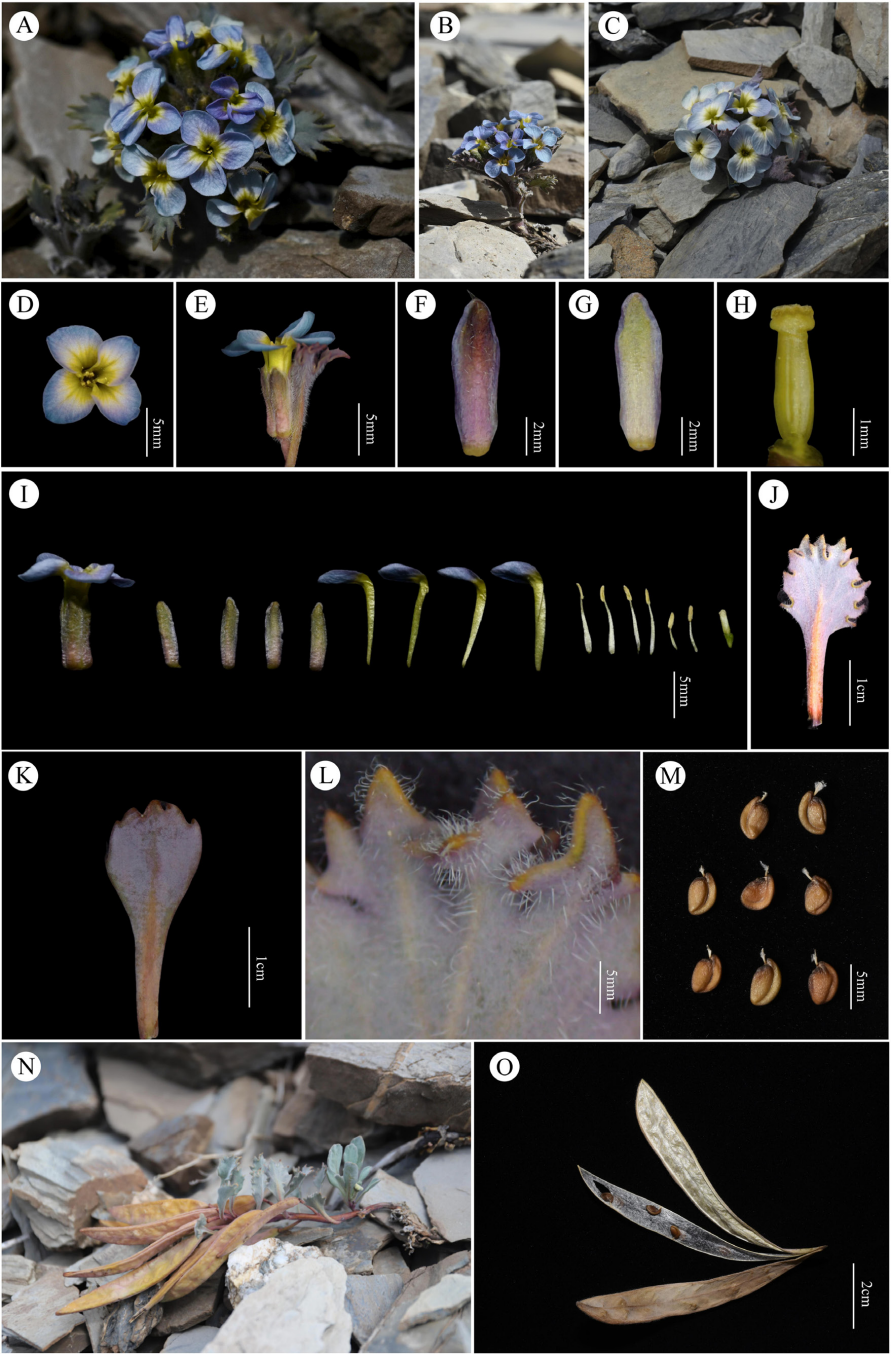
*Solms-laubachia garzeensis* J.Fu, Y.Ma & H.X.Yin, sp. nov. **A–C**. Flowering plant; **D**. Single flower; **E**. Single flower and bract leaves; **F**. Outer side of the Sepals; **G**. Inner side of the Sepals; **H**. Stigma and ovary; **I**. Floral morphology; **J**. Bract leaves; **K**. Basal leaves; **L**. Simple trichomes; **M**. Seeds; **N**. Plant at siliqueing stage; **O**. Silique.

**Figure 3.**
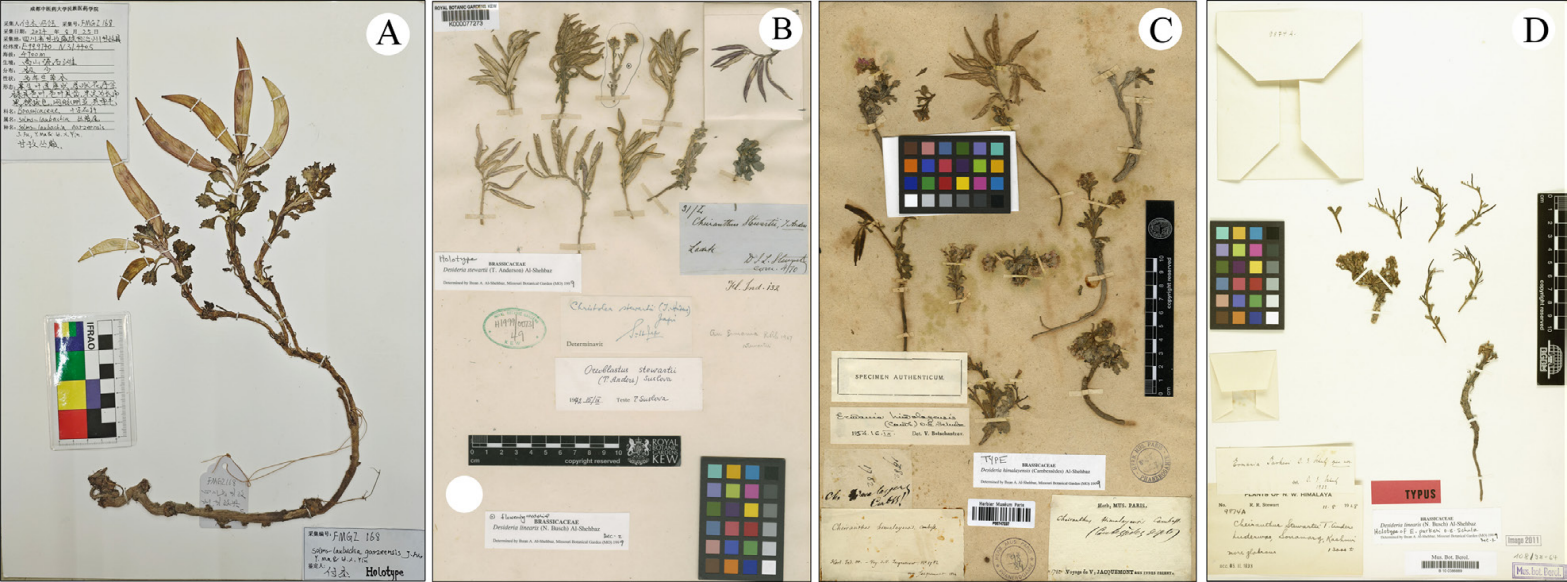
Specimens of four species. **A**. Holotype of *S. garzeensis* (Jie Fu and Yu Ma FMGZ168, CDCM!); **B**. Holotype of *S. stewartii* (J. L. Stewart s.n. K!); **C**. Lectotype of *S. himalayensis* (V. Jacquemont 1782, P!); **D**. Lectotype of *Ermania parkeri* (R. R. Stewart 9874A, B!) (*Ermania parkeri* is a synonym of *S. linearis*).

The morphological comparisons of the bract trichomes, leaves, flower color, siliques, and seeds are listed in Table 1, Fig. 1 and Fig. 2 displays the morphology of the new species along with detailed photographs. Fig. 3 presents images of the specimens for the new species and its three morphologically similar species.

**Table 1.** Morphological comparisons of *S. garzeensis*, *S. himalayensis*, *S. stewartii* and *S. linearis*.

Characters	*S. garzeensis*	*S. himalayensis*	*S. stewartii*	*S. linearis*
**Trichomes**	Trichomes simple, to 1.5 mm long	Trichomes simple, to 1.5 mm long	Trichomes stalked, forked, rarely simple, to 1.5 mm long	Trichomes simple, to 1.5 mm long
**Bracts**	Serrated shape on the margin	Entire	Entire	Entire
**Petal color**	Petals blue-violet, center yellowish–green	Petals purple or lilac with yellowish center	Flowers not seen	Petals purple or lavender with paler base
**Silique shape**	Silique oblanceolate	Silique lanceolate to lanceolate–linear	Silique lanceolate to lanceolate–linear	Silique linear
**Silique size**	3.7–11.1 cm × 7–14 mm	1.7–4.0 cm × 3.0–6.0 mm	1.7–3.5 cm × 3.0–5.0 mm	1.5–4.2 cm × 0.8–2.0 mm
**Seed size**	4.0–6.0 × 2.5–4.5 mm	1.5–2.3 × 1.0–1.4 mm	1.4–2.2 × 0.8–1.1 mm	0.8–1.1 × 0.5–0.8 mm

### Pollen grain morphology

**Figure 4.**
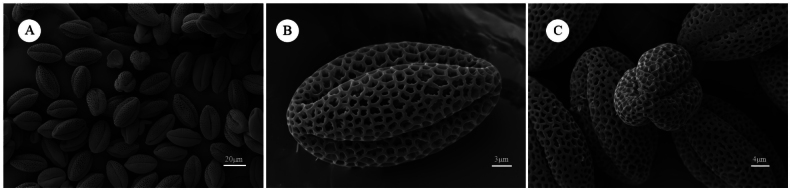
Pollen morphology of *Solms-laubachia garzeensis* under scanning electron microscopy. **A**. Pollen grain. **B**. Equatorial view. **C**. Polar view.

The pollen of *S. garzeensis* is tricolpate, and lobate-circular in polar view and is oblong in equatorial view. The pollen polar axis has a length of 33.82 μm, and the equatorial axis measures 17.99 μm, yielding a polar-to-equatorial axis ratio of 1.87. Small verrucous structures are observed within the pollen reticulum (Fig. 4). To date, Ying and Zhang (1994) have documented the pollen morphology of two species in the genus *S. ciliaris* and *S. linearifolia*. However, Al-Shehbaz and Yang (2001) later confirmed that *S. ciliaris* was based on material derived from *Leiospora pamirica*.

### Molecular phylogenetic analysis

**Figure 5.**
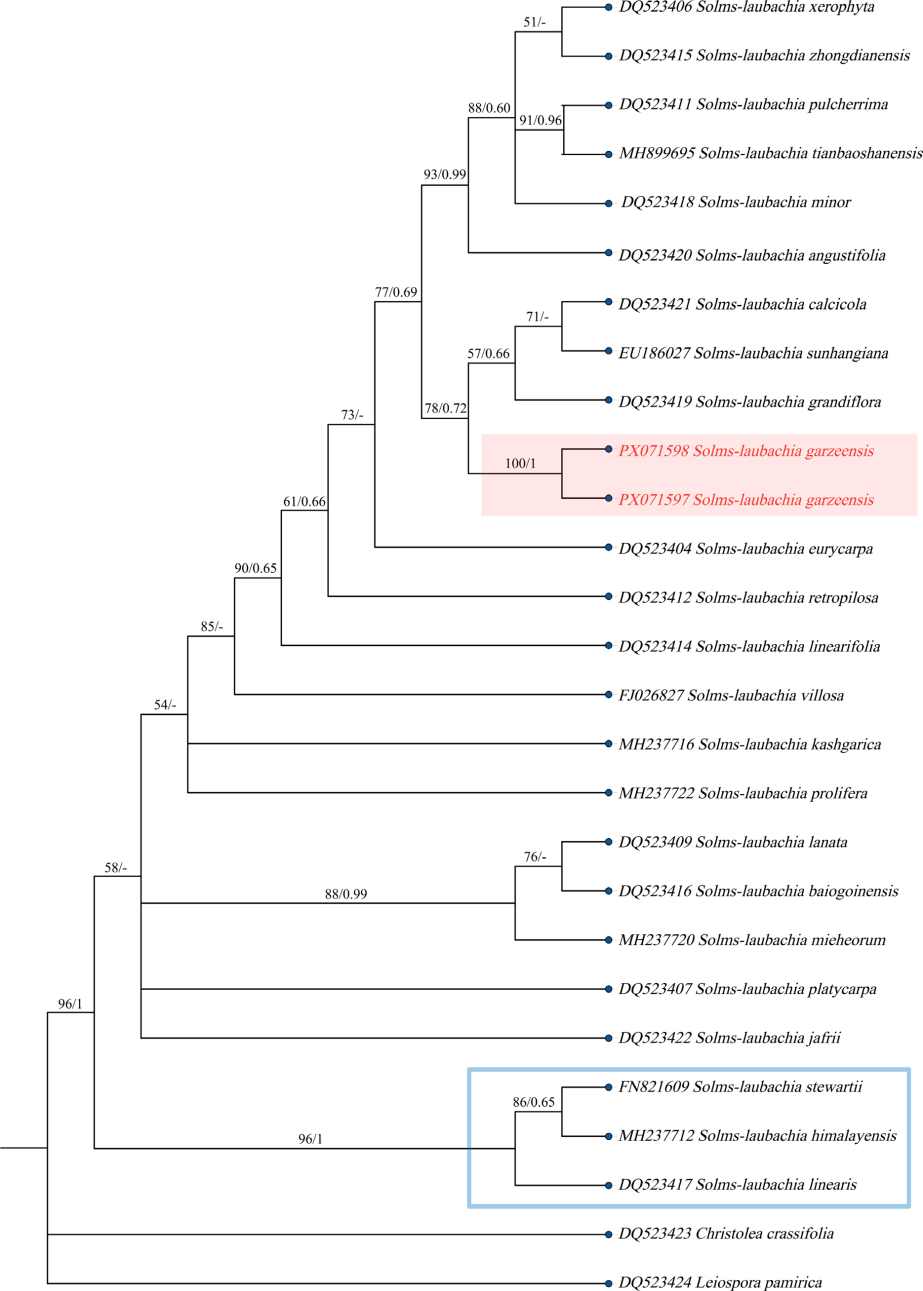
The ML phylogenetic tree of 24 *Solms-laubachia* species based on *nrITS* sequences is shown, Nodal support values are indicated as ML bootstrap percentages (BS) and Bayesian posterior probabilities (PP); dashes (-) denote nodes with BS < 50% or PP < 0.5, among which new species are shown in red, and closely related species are highlighted in blue boxes.

Maximum Likelihood (ML) and Bayesian Inference (BI) analyses produced similar tree topologies. The ML tree is depicted in Fig. 5, with bootstrap support (BS) values and Bayesian posterior probabilities (PP) indicated at nodes. The 24 species of *Solms-laubachia* form a well-defined monophyletic clade (BS = 96; PP = 1, Fig. 5). The two individuals of the new species cluster to form one clade (BS = 100; PP = 1, Fig. 5), and this new species is also grouped together with *S. grandiflora*, *S. calcicola*, and *S. sunhangiana* into a subclade (ML = 78; PP = 0.72, Fig. 5), and together with *S. angustifolia* and other species, six other species form a large clade (ML = 77; PP = 0.69 Fig. 5), along with *S. eurycarpa*, *S. retropilosa*, and *S. linearifolia*, they constitute the so-called HDM clade (Chen et al. 2018). Molecular systematics has demonstrated the uniqueness of the genetic characteristics of *S. garzeensis*, which strongly supports its status as a new species.

### Geographical distribution

**Figure 6.**
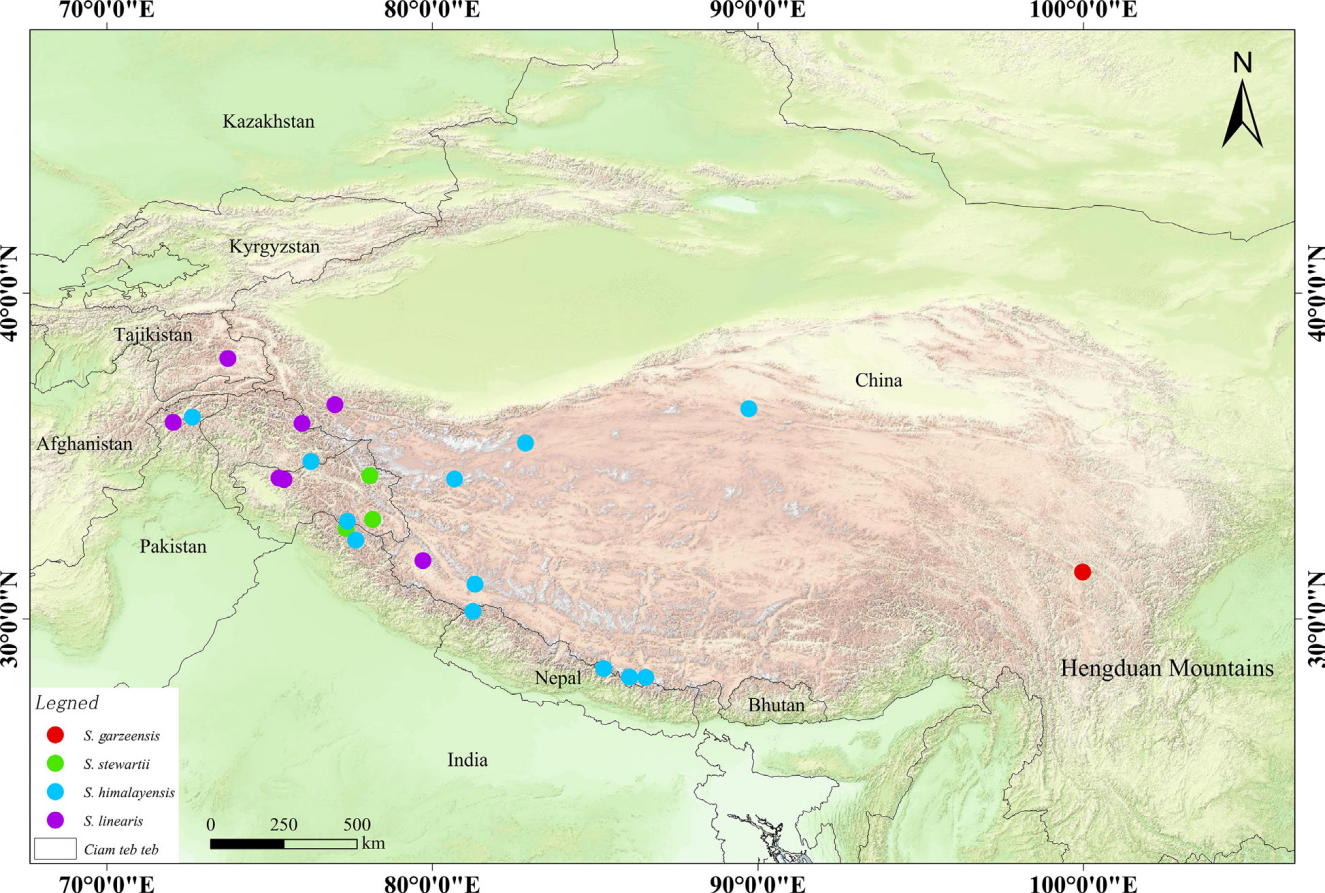
The geographical distributions (collated from specimen data) of *S. garzeensis*, *S. himalayensis*, *S. stewartii* and *S. linearis*.

*S. himalayensis*, *S. stewartii*, *S. linearis*, and the newly described *S. garzeensis* exhibit distinct geographical distributions. Based on specimen records and literature, *S. himalayensis* has the widest distribution, ranging northward from the Himalayas to the Kunlun Mountains, encompassing Nepal, India, northern Pakistan, as well as Tibet and southern Xinjiang in China, but notably excluding the Hengduan Mountains region (Fig. 6). The distribution of *S. linearis* overlaps partially with that of *S. himalayensis*. However, *S. linearis* is concentrated in the Pamir Plateau and also distributed in the western Himalayas, including eastern Tajikistan, northeastern Pakistan, as well as southwestern Xinjiang and western Xizang of China, with no occurrence in the Hengduan Mountains region (Fig. 6). *S. stewartii* is regarded by scholars as an extremely rare and uncommon species (Lan 1987; Al-Shehbaz 2000). Preliminary assessments from existing specimen records suggest a distribution overlapping of the aforementioned species, although this requires further clarification.

Based on available evidence, some specimens from China previously identified as *S. stewartii* are in fact *S. himalayensis*, consistent with the view of Al-Shehbaz (2000). Therefore, the actual distribution of *S. stewartii* is likely narrower than current records suggest. Based on type specimens, Kashmir and adjacent areas represent more reliable distribution ranges for *S. stewartii* (Fig. 6). To date, none of the three compared species have been recorded in the Hengduan Mountains, showing clear geographical isolation from *S. garzeensis*. This pattern may reflect high species diversification in *Solms-laubachia* driven by Quaternary glacial-interglacial dynamics.

### Taxonomy

#### Solms-laubachia garzeensis

Taxon classificationPlantaeAsparagalesAsparagaceae

 J.Fu, Y.Ma & H.X.Yin, sp. nov.

1756AD8F-0EA1-5177-B52D-BEF61A7E70D5

urn:lsid:ipni.org:names:77378626-1

##### Diagnosis.

**Figure 7.**
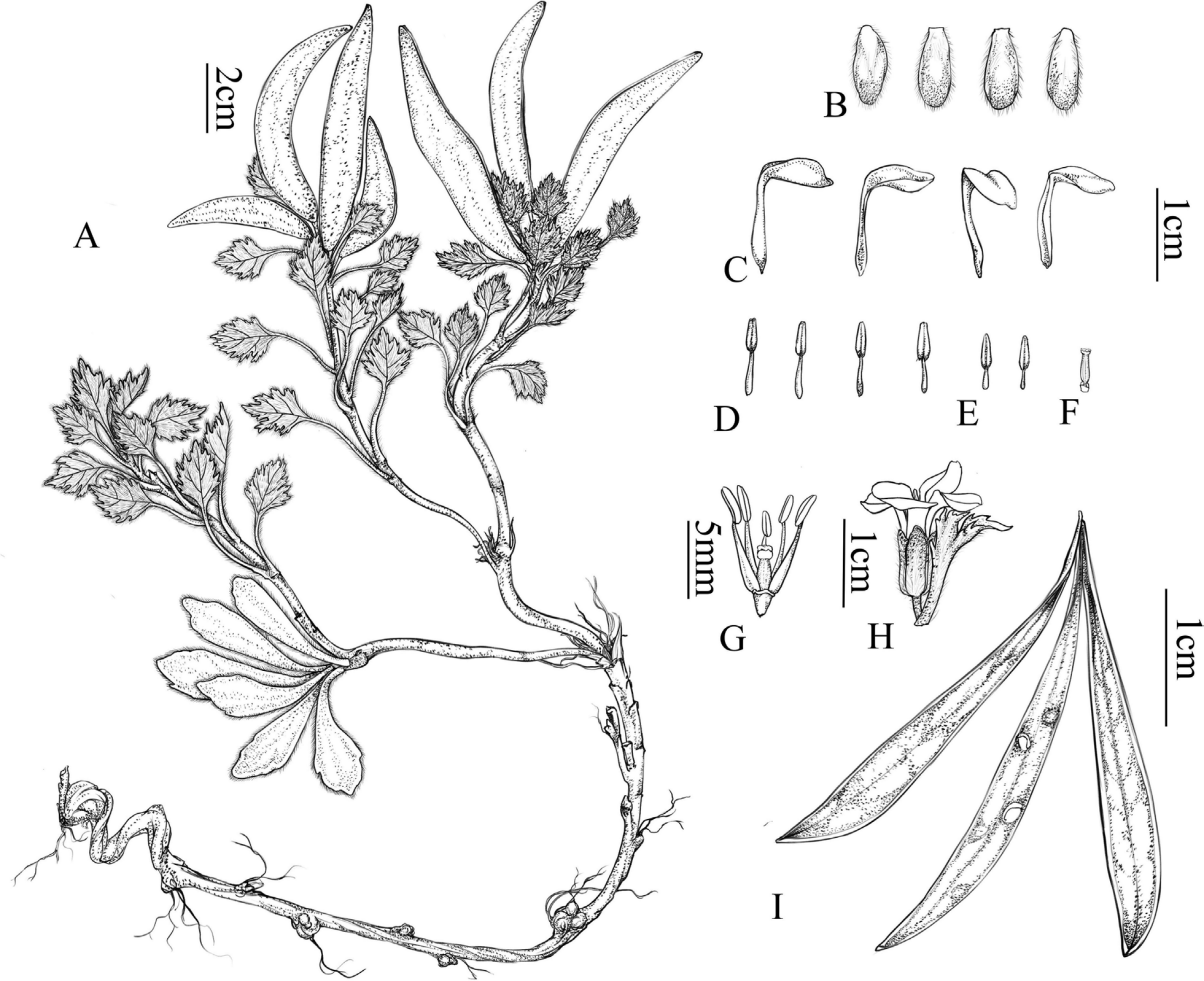
*Solms-laubachia garzeensis* J.Fu, Y.Ma & H.X.Yin, sp. nov. **A**. Habit; **B**. Sepal; **C**. Petal; **D**. Inner stamen; **E**. Outer stamen; **F**. Pistil; **G**. Stamens and pistil; **H**. Flower and bracts; **I**. Silique. Drawn by Ms. Changyun Dai.

*S. garzeensis* is morphologically similar to *S. himalayensis*, *S. stewartii* and to *S. linearis*. All four species have rosette-shaped, hairy basal leaves, racemes with bracts throughout the plant, and long, readily cracked and detached siliques. However, by synthesizing the characteristics of simple trichomes, blue-violet petals, large siliques, toothed bracteal leaves and cryptic coloration possessed by the new species, it can be distinctly distinguished from the aforementioned three species (An ink-line drawing of *S. garzeensis* is presented in Fig. 7).

##### Type.

**China • Sichuan Province**: Garze County, Shaluli Mountains, on scree slopes, 31°26.43'N, 99°58.44'E, elev. ca. 4700 m, 10 September 2024, *Jie Fu & Yu Ma* FMGZ168 (***holotype***: CDCM!; ***isotype***: CDBI!, KUN!).

##### Description.

***Perennial herbs*** 1.8–6 cm tall, densely pilose throughout with simple trichomes to 1.5 mm long. ***Stems*** 4–10 cm tall, simple, pilose. ***Basal leaves*** fleshy, grayish-green to brownish gray, densely pilose, persistent; petiole 0.5–2.4 cm long, ciliate; leaf blade spatulate or flabellate, 14–25 × 6–12 mm, base cuneate to attenuate, margin with 3–6 teeth, apex acute, rarely entire. ***Racemes*** 4–25 flowered, bracteate throughout; bracts similar to basal leaves but smaller, adnate to pedicel, with 6–13 sharp teeth; siliqueing pedicels ascending, straight or curved, pilose. ***Sepals*** free, oblong, 7–10 × 2.5–4 mm, caducous, pilose, margin purple-membranous, base not saccate. ***Petals*** blue-violet with yellowish-green center, 4.5–6 mm long, 5–7 mm wide; claw 8–10 mm long; limb obovate to broadly so. ***Stamens*** median filaments 5–8 mm long, lateral filaments 3–5 mm long; anthers 1.8–2.5 mm long. ***Silique*** lanceolate, 3.7–11.1 cm × 7–14 mm, strongly flattened; valves pilose or glabrous, distinctly veined; septum complete, membranous; style obsolete; stigma 2-lobed. ***Seeds*** claybank, ovate, biseriate, 4–6 × 2.5–4.5 mm.

##### Phenology.

Flowering period May–June; siliqueing period September–October.

##### Etymology.

**Figure 8.**
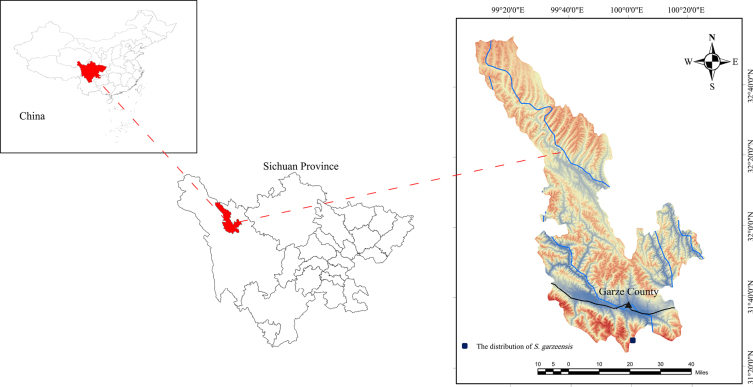
Location of the distribution of *S. garzeensis* in Garze, Sichuan.

This species was discovered in the Shaluli Mountains located at the border between Garze County and Xinlong County (Fig. 1 and Fig. 8). The Shaluli Mountains stretch continuously here, with the highest altitude exceeding 5900 m. Garze means “the place favored by the Bodhisattva” in Tibetan. This is an important economic and cultural center in the northern part of the Kham Tibetan region. The name of this species comes from Garze County, where it was discovered. “Garze” is a transliteration of the Tibetan term “དཀར་མཛེས”, and it is currently the internationally accepted name for this region.

##### Distribution and habitat.

After years of field survey on HDM, up to now, we have only discovered this species in the Shaluli Mountains at the border of Garze County and Xinlong County. It grows on gravel slope areas at an altitude of approximately 4700 meters (Fig. 1), and its associated plants include *Saussurea medusa*, *Bistorta vivipara*. Currently, *S. garzeensis* is facing threats such as a gradually shrinking distribution area and a decline in population due to human activities. Due to global warming and human disturbance, the habitat and distribution range of *S. garzeensis* have been steadily diminishing. Concurrently, due to pronounced geographical isolation, the population faces a potential risk of inbreeding depression.

Applying the IUCN Red List Categories and Criteria (Version 16.0, IUCN Standards and Petitions Committee 2024), our assessment demonstrates that the results are consistent with [B1ab (i, ii, iii) ]and (C1), extent of occurrence (EOO) < 100 km^2^ (B1), severely fragmented habitat (a), with continuing decline (b) in extent of occurrence (i), area of occupancy (ii) and area, extent and/or quality of habitat (iii). The number of mature individuals within this population is less than 250 and continues to decline (C1). We provisionally assess it as Critically Endangered (CR).

##### Additional specimens examined (paratypes).

The same locality as holotype, May 29, 2025, *Jie Fu & Yu Ma*, FJMYGZ201 (CDCM! , KUN!).

## Discussion

The survival conditions in the alpine subnival belt above 4000 meters altitude are extremely harsh. To reduce the probability of being foraged by animals, plants have developed a special survival skill known as cryptic coloration. Typical examples include *Fritillaria delavayi* (Huang et al. 2024) and *Corydalis hemidicentra* (Niu et al. 2017). The unique cryptic coloration of *S. garzeensis* also serves the same function, making the plants extremely difficult to detect during their growth periods except for the florescence. This phenomenon has been reported for the first time in the genus *Solms-laubachia*. The unique cryptic coloration of *S. garzeensis* is a key reason why it has only been discovered up to now.

In the nuclear (*nrITS*)-based phylogenetic tree, *S. garzeensis* was nested within the Hengduan Mountains (HDM) clade and formed a moderately supported subclade with *S. grandiflora*, *S. calcicola*, and *S. sunhangiana*. All four species are HDM endemics, restricted to its specific native habitat respectively (Yue et al. 2009b). Obviously, the molecular phylogenetic results are highly consistent with the geographical distribution characteristics, which reflects a close genetic affinity among these species. Chen (2019) proposed several potential new taxa distributed in the HDM and also suggested the possible existence of a *S. sunhangiana*-*S. grandiflora* species complex with close genetic relationships. The molecular phylogenetic results combined with plant geographical characteristics of this research indicating that the new species may also belongs to the potential species complex proposed by Chen. The vertical altitudinal variation in the Hengduan Mountains (HDM) is far more pronounced than in other regions, leading to exceptionally strong speciation and geographical isolation effects. This region harbors the highest intra-generic species diversity (Yue 2006; Chen 2019). Within the HDM clade, most members belong to the traditionally defined *Solms-laubachia* lineage, characterized by linear, lanceolate, or oblanceolate leaves with entire margins. *S. garzeensis* is currently the only species that possesses toothed leaf margins, palmate venation, and distinctive cryptic coloration in the HDM clade. Yue et al. (2009a) suggested that the genus is still undergoing rapid and active evolution, likely influenced by reticulate evolution, recombination, and incomplete lineage sorting. Therefore, we speculate that all members of the HDM clade may have originated from a common ancestor and undergone adaptive evolution in their respective unique habitats, resulting in significant morphological differences among them. However, this hypothesis awaits verification by more extensive and comprehensive phylogenetic research. *S. garzeensis* can be regarded as representative of the high morphological and ecological diversity of *Solms-laubachia* in the HDM region. Together, its unique morphology and phylogenetic placement provide strong evidence for the recognition of *S. garzeensis* as a distinct new taxonomic entity.

### Key to related species

**Table:** 

1a	Leaves dentate; racemes with bracts throughout the plant	**2a**
2a	Bract leaves with 6–13 teeth on the margins, silique lanceolate	**3a**
3a	Petals blue-violet with yellowish-green center, silique 3.7–11.1 cm long, 7–14 mm wide, seeds 4–6 mm long, 2.5–4.5 mm wide, plants with simple trichomes only	**1. *S. garzeensis***
2b	Bracts entire, siliques lanceolate to linear–lanceolate	**4a**
4a	Plants with simple trichomes only	**5a**
5a	Silique less than 3 mm wide	**6a**
6a	Silique 0.8–2.0 mm wide, seeds 0.8–1.1 mm long, 0.5–0.8 mm wide	**3. *S. linearis***
5b	Silique more than 3 mm wide	**6b**
6b	Silique 3.0–6.0 mm wide, seeds 1.5–2.3 mm long, 1–1.4 mm wide	**2. *S. himalayensis***
4b	Plants with simple trichomes and trichomes stalked forked	**4. *S. stewartii***

## Supplementary Material

XML Treatment for Solms-laubachia garzeensis

## Additional information

### Use of AI

No use of AI was reported.

### Author ORCIDs

Jie Fu https://orcid.org/0009-0009-6084-5279

Yu Ma https://orcid.org/0009-0003-9954-3872

Zhongyi Zhao https://orcid.org/0009-0001-7005-1677

Hongling Chen https://orcid.org/0009-0005-5091-7655

Hongxiang Yin https://orcid.org/0000-0003-1411-6365

### Data availability

The sequences used in this study have been deposited in the GenBank database (https://www.ncbi.nlm.nih.gov/genbank) under accession numbers PX071597 and PX071598.

## Supplementary materials

Supplementary material 1 Appendices Data type rtf Explanation note **appendix S1**. Species used in the molecular system analysis of this article and GenBank accession numbers for (nrITS) sequences. **appendix S2**. Specimens of related species examined. https://binary.pensoft.net/file/1595331This dataset is made available under the Open Database License (http://opendatacommons.org/licenses/odbl/1.0/). The Open Database License (ODbL) is a license agreement intended to allow users to freely share, modify, and use this Dataset while maintaining this same freedom for others, provided that the original source and author(s) are credited. Jie Fu, Yu Ma, Zhongyi Zhao, Hongling Chen, Hongxiang Yin
