# Corrigendum: Fu J, Ma Y, Zhao Z, Chen H, Yin H (2026) *Solms-laubachia garzeensis* (Brassicaceae), a new species from the Hengduan Mountains of Sichuan Province, China. PhytoKeys 273: 93–107.https://doi.org/10.3897/phytokeys.273.177843

**DOI:** 10.3897/phytokeys.278.202470

**Published:** 2026-08-25

**Authors:** Jie Fu, Yu Ma, Zhongyi Zhao, Hongling Chen, Hongxiang Yin

**Affiliations:** 1 College of Ethnomedicine, Chengdu University of Traditional Chinese Medicine, Chengdu 611137, China College of Ethnomedicine, Chengdu University of Traditional Chinese Medicine Chengdu China; 2 School of Pharmacy, Chengdu University of Traditional Chinese Medicine, Chengdu 611137, China School of Pharmacy, Chengdu University of Traditional Chinese Medicine Chengdu China

In our recent work (Fu et al. 2026), the species key presented in the publication does not follow the standard dichotomous format widely adopted in plant taxonomy. Here, we provide a revised and taxonomically correct species key.

## Key to related species

**Table:** 

1	Bract leaves with 6–13 teeth on the margins, plants with simple trichomes only, siliques 7.0–14.0 mm broad	**1. *S. garzeensis***
–	Bracts entire, siliques ≤ 6.0 mm wide	**2**
2	Plants with simple trichomes and trichomes stalked forked	**2. *S. stewartii***
–	Plants with only simple trichomes	**3**
3	Siliques 0.8–2.0 mm wide, seeds 0.8–1.1 mm long, 0.5–0.8 mm wide	**3. *S. linearis***
–	Siliques 3.0–6.0 mm wide, seeds 1.5–2.3 mm long, 1.0–1.4 mm wide	**4. *S. himalayensis***

## Additional information

### Artificial Intelligence (AI) use

The authors accept full responsibility for the content of the manuscript, including the disclosure of any use of AI.

No AI tools were used in the preparation of this manuscript.

### Author ORCIDs

J. Fu https://orcid.org/0009-0009-6084-5279

Y. Ma https://orcid.org/0009-0003-9954-3872

Z. Zhao https://orcid.org/0009-0001-7005-1677

H. Chen https://orcid.org/0009-0005-5091-7655

H. Yin https://orcid.org/0000-0003-1411-6365

### Data availability

All of the data that support the findings of this study are available in the main text.
