# Supplementary material for: Solms-laubachia garzeensis (Brassicaceae), a new species from the Hengduan Mountains of Sichuan Province, China
Source: PhytoKeys. 2026 Apr 15;273:93–107. doi: 10.3897/phytokeys.273.177843 (PMC13103667; doi:10.3897/phytokeys.273.177843)
Supplement: Supplementary material 1 — Appendices [file phytokeys-273-093_article-177843__-s001.rtf]

Appendix S1
Species used in the molecular system analysis of this article and GenBank accession numbers for (nrITS) sequences:
 Leiospora pamirica (Botsch. & Vved.) Botsch. & Pachom. (DQ523424); Christolea crassifolia Cambess. (DQ523423); S. garzeensis J. Fu, Y. Ma & H. X. Yin.(1)(PX071598, PX071597); S. himalayensis (Cambess.) J. P. Yue, Al-Shehbaz & H. Sun (FN821208); S. stewartii (Anderson), J. P. Yue, Al-Shehbaz & H. Sun (FN821609); S. linearis (N. Busch) J. P. Yue, Al-Shehbaz & H. Sun (DQ523417); S. mieheorum (Al-Shehbaz) J. P. Yue, Al-Shehbaz & H. Sun (MH237720); S. baiogoinensis (K.C. Kuan & C.H. An) J. P. Yue, Al-Shehbaz & H. Sun (DQ523416); S. tianbaoshanensis H.L. Chen, Al-Shehbaz, J. P. Yue & H. Sun (MH899695); S. grandiflora J. P. Yue, Al-Shehbaz & H. Sun (DQ523419); S. sunhangiana J. P. Yue & Al-Shehbaz (EU186027); S. angustifolia J. P. Yue, S. calcicola J. P. Yue, Al-Shehbaz & H. Sun (DQ523421); S. xerophyta (W.W. Sm.) Comber (DQ523406); S. zhongdianensis J. P. Yue, Al-Shehbaz & H. Sun (DQ523415); S. minor Hand.-Mazz. (DQ523418); S. pulcherrima Muschl. (DQ523411); S. retropilosa Botsch. (DQ523412); S. lanata Botsch. (DQ523409); S. villosa (Maxim.) D. German & Al-Shehbaz (FJ026827); S. eurycarpa (Maxim.) Botsch. (DQ523404), Al-Shehbaz & H. Sun (DQ523420); S. linearlifolia (W.W. Sm.) O. E. Schulz (DQ523414); S. platycarpa (Hook.f. & Thomson) Botsch. (DQ523407); S. prolifera (Maxim.) J. P. Yue, Al-Shehbaz & H. Sun (MH237722); S. jafrii (Al-Shehbaz) J. P. Yue, Al-Shehbaz & H. Sun (DQ523422); S. kashgarica (Botsch.) D. German & Al-Shehbaz (MH237716). 

Appendix S2 Specimens of related species examined:
S. himalayensis
CHINA. Tibet Autonomous Region: W Tibet, in declivitate orientali jugi vulgo Kioubrung-ghauti in Tartaria sinensi, 1834, V. Jacquemont 1782 (isotype: K [K000774321]!). Baigoin County: on alpine tundra, 4900–5200 m, 24 Aug 1988, S. G. Wu et al. 4075 (HNWP!, KUN!, PE!). Geji County: Near Mount Alin, 5300 m, 15 Aug 1976, Qinghai-Xizang Expedition Team 76-8652 (HNWP!, PE!). Pulan County: near Mount Kailash, 5100–5300 m, 24 Aug 1974, Xizang Expedition Team of the Institute of Biology 4024 (PE!, HNWP!). Ritu County: Duoma, Quezheredu, 5600 m, 15 Aug 1976, Qinghai-Xizang Expedition Team 76-9061 (KUN!, HNWP!); south of Longmu Lake, 15 Aug 1976, Qinghai-Xizang Expedition Team 76-12961 (PE!). Shuanghu County: Mount Mayil, 5400–5500 m, 25 Aug 1976, Qinghai-Xizang Expedition Team, Lang K.Y. 10059 (KUN!). Xinjiang Uygur Autonomous Region: Ruoqiang County: northeast of Whale Lake, south of the main ridge of Kunlun Mountain, 15 Aug 1988, Zhen D., Zhang L.D., Guo K. 12450 (PE!).
NEPAL. Gandaki Province: Dhaulagiri Himal, Hidden Valley, between Dhampus Pass and French Pass, 5075–5230 m, 1980, Women's Climbing Expedition 65 (BM!).
INDIA. Jammu & Kashmir, Ladakh: among dense pile of broken rocks, 5335 m, 19 Aug 1931, Walter N. Koelz 2667 (NY!). Leh District: Taklung La, 5182 m, 14 Aug 1933, Walter N. Koelz 6500 (MICN!).
PAKISTAN. Khyber Pakhtunkhwa Province: Chitral District: Barum Gol, above Jamishi Ghochar, 4300 m, 16 Jul 1950, Per Wendelbo & Finn Jorstad s.n. (O!).
S. stewartii
CHINA. Tibet Autonomous Region: boreali-occidentalis, in altoplanitie tibetana, prope camp 463, 4900 m, 15 Aug 1939, Nils Ambolt S-GH-4305 (S!).
INDIA. Jammu & Kashmir, Ladakh: [locality details not specified], 4572–5029 m, J. L. Stewart s.n. [holotype: K (K000774322!); isotype: E!]. Himachal Pradesh: Lahaul and Spiti District: Baralachala, Lahul, Kangra, Punjab, 5030 m, 9–13 Jul 1933, Walter N. Koelz 5375 (MICN!); 5030 m, 26–29 Aug 1933, Walter N. Koelz 6773a (MICN!); 5060 m, 25 Aug 1916, A. K. Bulley 5490 (P!).
S. linearis
CHINA. Tibet Autonomous Region: Zhada County: Laozhishankou, 5200 m, 6 Jul 1976, Qinghai-Xizang Expedition Team 7948 (PE!, HNWP!). Xinjiang Uygur Autonomous Region: Yeicheng County: Kunlun Mountains, 5550 m, 25 Aug 1987, B. S. Li et al. 4278 (PE!). Pishan County: Mazhada Ban, 5010 m, 13 Jul 2017, Yue Jipei & Chen Hongliang YC-XZ123 (KUN!).
INDIA. Jammu & Kashmir: Sonamarg, Luderwas, 3960 m, 11 Aug 1928, R. R. Stewart 9874A [lectotype: B (B100246777!)]. Sind Valley: 3200 m, 2 Sep 1982, G. H. Da 3934 (MO!).
PAKISTAN. Gilgit-Baltistan: Karakoram, earthy places, Blation Valley, 4750 m, 28 Jul 1967, O. Polunin 6133 (BM!). Khyber Pakhtunkhwa Province: Chitral District, Laspur (Hachin), 4420 m, 1958, S. Bowes Lyon 44 (USNM!).
TAJIKISTAN. Gorno-Badakhshan Autonomous Region: Pamir, Schugnan Abchary, 2 Aug 1904, B. Fedtschenko s.n. (holotype: LE!).
